# Structural Configuration of Blood Cell Membranes Determines Their Nonlinear Deformation Properties

**DOI:** 10.1155/2022/1140176

**Published:** 2022-04-18

**Authors:** Elena Kozlova, Viktoria Sergunova, Vladimir Inozemtsev, Ekaterina Sherstyukova, Aleksandr Kozlov, Olga Gudkova, Aleksandr Chernysh

**Affiliations:** ^1^Laboratory of Biophysics of Cell Membrane under Critical State, Federal Research and Clinical Center of Intensive Care Medicine and Rehabilitology, V.A. Negovsky Research Institute of General Reanimatology, Moscow 107031, Russia; ^2^Department of Medical and Biological Physics, Sechenov First Moscow State Medical University (Sechenov University), Moscow 119991, Russia; ^3^Faculty of Physics, Federal State Budget Educational Institution of Higher Education M.V. Lomonosov Moscow State University (Lomonosov MSU), Moscow 119234, Russia

## Abstract

The ability of neutrophils and red blood cells (RBCs) to undergo significant deformations is a key to their normal functioning. Disruptions of these processes can lead to pathologies. This work studied the influence of structural configuration rearrangements of membranes after exposure to external factors on the ability of native membranes of neutrophils and RBCs to undergo deep deformation. The rearrangement of the structural configuration of neutrophil and RBC membranes under the influence of cytological fixatives caused nonlinear deformation phenomena. There were an increase in Young's modulus, a decrease in the depth of homogeneous bending, and a change in the distance between cytoskeletal junctions. Based on the results of the analysis of experimental data, a mathematical model was proposed that describes the process of deep bending of RBСs and neutrophil membranes.

## 1. Introduction

Blood cells in the process of circulation in the vascular bed undergo significant deformations. Red blood cells (RBCs) must maintain elasticity to pass through capillaries [1, 2], and neutrophils must leave through the walls of blood vessels into tissues [3–5]. The passage of blood cells through a narrow space is critical for their normal functioning [2, 3]. An increase in cell stiffness can make it difficult for RBCs to pass into capillaries [6, 7] and for neutrophils to migrate through tissue (gaps) [8–10]. Often, an increase in the membrane stiffness and a loss of deformability are associated with malaria, sickle cell anemia, diabetes, spherocytosis, myocardial infarction, sepsis, and prolonged storage of donor blood [9, 11–18].

The biomechanical properties of blood cells are determined by the stiffness of their membranes and the state of the cytoskeleton lining on the inner side of the cell [19]. In neutrophils, this lining is a complex of an actin network with microtubules that are associated with the membrane [20–23], and in RBCs, the lining is a network of spectrin filaments associated with a lipid layer by band 3 proteins at the spectrin-ankyrin binding sites and actin junctional complexes [24, 25]. The structural configuration of the membranes of even nonactivated neutrophils is much more complicated than that of RBCs. The membrane of neutrophils over the entire surface has wrinkles [26–28], protrusions, and lamellipodia, providing cell motility. The RBC membrane is almost smooth, without folds. A large number of scientific studies have been devoted to the study of the causes and mechanisms of disturbances of the elastic properties of RBC membranes [29–31]. The elastic properties of neutrophil membranes have been studied less. These properties are determined by the great variability of the morphology of individual neutrophils, even in an inactivated state.

In most work, the stiffness of the membranes is estimated by the value of Young's modulus using the classical Hertz model. This approach assumes the whole cell as a homogeneous, linear, isotropic material [32–36]. Such measurements, as a rule, are held at depths of probe indentation at up to 0.1–0.3 of the cell height (up to 300–400 nm). However, recent studies show that deformation properties depend not only on the intrinsic properties and features of the membranes but also on the values of their deformations [37]. These phenomena occur at depths of 0.6–0.7 of the cell height. In other words, the biomechanical characteristics and, in particular, Young's modulus can change during the process of deep bending of the membrane. Currently, the parameters and quantitative estimates of the zones for which the Hertz model can be adequately applied and the depths to which bending is conducted in a homogeneous environment remain insufficiently studied. It is not clear how the membranes behave at depths of large homogeneous bending zones or how nonlinear phenomena appear themselves at such depths (>1000 nm).

In our *in vitro* work, we studied how the deformation characteristics of native neutrophils and RBCs change with an increase in the bending depth of their membranes and how nonlinear phenomena of the slowdown of probe indentation arise. Specifically, we investigated the relationships between changes in the structural configuration of the membranes of nonactivated neutrophils/RBCs and the nonlinear characteristics of bending in these cells, and we built a model that describes the nature of the nonlinearity of the biomechanical properties of membranes.

To change the structural configuration and the stiffness of the membranes, we used fixatives that are widely used in practice—methanol and glutaraldehyde. To measure the biomechanical characteristics of membranes, we used atomic force spectroscopy. To describe nanosurfaces and structural configurations, we used atomic force and laser confocal fluorescence microscopy [38, 39]. The parameters of the cytoskeleton networks were determined using the nearest neighbor distance method.

## 2. Materials and Methods

### 2.1. The Scheme of Experiments

**Figure 1:**
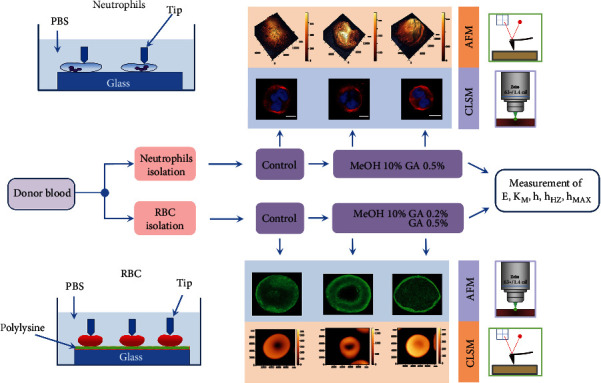
Structural configuration and deformation characteristics of deep bending of membranes under the action of modifiers. Schemes for conducting experiments on the membranes of neutrophils and RBCs. AFM: atomic force microscope; CLSM: confocal laser scanning microscopy; GA: glutaraldehyde; MeOH: methanol; tip: AFM cantilever probe; PBS: buffer solution.

Experiments in vitro were conducted according to the scheme shown in Figure 1.

At the first stage of the experiment, suspensions of neutrophils and RBCs were prepared. The suspensions were prepared from the blood of 5 healthy volunteers of both sexes, aged 25 to 40 years.

In the second stage, during the experiment, modifiers (fixators) were added to RBCs and neutrophils.

### 2.2. Isolation of Human Neutrophils

The study used venous blood from 5 healthy volunteers of both sexes, 25 to 40 years of aged. Neutrophils were isolated according to the standard protocol for blood density gradient separation using Ficoll solutions [40].

The blood was separated on a double density gradient of Ficoll *ρ* = 1.119 g/cm^3^ and *ρ* = 1.077 g/cm^3^ (Paneco, Russian Federation) and centrifuged with no braking (1000 rpm, 40 minutes) by a Universal 320 centrifuge (Andreas Hettich GmbH & Co. KG, Germany). Then, the bottom cloudy white ring was collected. Neutrophils were washed three times by centrifugation (1000 rpm, 5 minutes) with phosphate-buffered saline (PBS) without calcium and magnesium (MP Biomedicals, France). Then, the cells were put on the glass surface and left to settle at room temperature for 1 h.

Settled neutrophils were exposed to 0.5% glutaraldehyde and 10% methanol for 4 minutes. The resulting samples were washed with PBS. The cell purity was >95% of the isolated cells (without RBCs).

### 2.3. Isolation of Human Red Blood Cells. RBC Suspensions

Blood sampling was conducted in microvettes with EDTA (Sarstedt AG and Co., Germany) during a prophylactic examination from 5 donors.

Then, 150 *μ*l of fresh human blood was centrifuged at 3000 rpm for 5 minutes to separate the blood cells from the plasma. The plasma was removed, and the volume was brought to that of the original blood sample with PBS. Hence, in suspension, the RBC concentration was the same as that in the initial blood. The RBCs were washed three times in PBS. In the second stage of the experiment, various modifiers were added to RBCs.

### 2.4. Preparation of Chemical Agents

Glutaraldehyde solution (25%) (Panreac Quimica S.L.U., Spain) was used to prepare the working solutions at 0.5% and 0.2%. Methanol HPLC (Chimmed, Russian Federation) was used to prepare the working solution at 10%. Glutaraldehyde or methanol was added to the cell suspension at a volume ratio of 1 : 1. These suspensions were marked correspondingly as GA0.2 and GA0.5 and MeOH10. The cell suspension was incubated for up to 4 minutes.

### 2.5. Fluorescence Staining of Neutrophils

After fixation, the samples were washed in PBS 3 times for 5 min. Then, cells were permeabilized with TritonX-100 0.05% (Sigma, USA) for 15 min. DNA was stained with Hoechst 33342 dye (Sigma, USA) at a ratio of 1 to 1000 for 20 min. For neutrophil plasma membrane staining, we used Alexa Fluor 594-labeled wheat germ agglutinin (WGA) (Thermo Fisher Scientific, USA) (1 : 500 for 20 min in PBS). Alexa Fluor 488-labeled phalloidin (Thermo Fisher Scientific, USA) was used to stain F-actin (1 : 600 for 20 min in PBS). The dyes with the samples during staining were kept in the dark. After staining, coverslips were washed 3 times in PBS and then mounted using Abberior Mount Solid Antifade (Abberior, Germany) on slides before imaging.

### 2.6. Fluorescence Staining of RBCs

To localize F-actin in RBCs, smears were prepared on a coverslip using the V-Sampler (Vision, Austria). After fixation, the samples were washed and permeabilized as described before. Alexa Fluor 488-labeled phalloidin (Thermo Fisher Scientific, USA) was used to stain F-actin (1 to 600 for 20 min in PBS in the dark). After staining, coverslips were washed 3 times in PBS and then mounted using Abberior Mount Solid Antifade (Abberior, Germany) on slides before imaging.

### 2.7. Confocal Laser-Scanning Microscope

For excitement with Hoechst 33342, a 405 nm laser was used; to excite Alexa Fluor 488 phalloidin, a 488 nm laser was used; to excite Alexa Fluor 594 WGA, a 543 nm laser was used.

Images were acquired with a Zeiss LSM880 confocal laser scanning microscope (Carl Zeiss, Germany) with the Airyscan module using a 63x oil immersion lens.

### 2.8. Methods of Analyzing Fluorescence Images

The resulting images were processed in the program ImageJ (Rasband, W.S., ImageJ, National Institutes of Health, USA). To estimate the distance between the actin components of the cytoskeleton, fast Fourier transform was performed, and the distance between the nearest fluorescence maxima (nearest neighbor distance) was determined using Find Maxima tool. Colocalization analysis was performed using JACoP plugin [41]. Colocalization is the spatial coincidence in one place of two or more fluorescent labels of different wavelengths, which makes it possible to quantify the interaction between the objects under study. In this work, to assess colocalization, the Manders coefficients and the Pearson coefficient were used [42, 43].

The Manders coefficient *M*_1_ is the ratio of the number of pixels of color channel 1 colocalized with pixels of channel 2 relative to the total number of pixels in channel 1. It describes cooccurrence—the proportion of one protein that colocalizes with another. It takes values from 0 to 1, where 0 is the absence of colocalization of the pixels of different channels and 1 is the complete spatial coincidence of the channel pixels.

The Pearson correlation coefficient estimates the correlation between two channels and reflects the linear relationship between the signal intensities of different color channels. Values can range from 1 (perfect positive correlation) to -1 (perfect negative correlation), and 0 means no correlation.

### 2.9. Atomic Force Microscopy

Cell images were obtained using an AFM NTEGRA Prima (NT-MDT Spectrum Instruments, Russian Federation) in tapping mode. Cantilevers NSG01 (TipsNano, Estonia) were used with the manufacturer-provided parameters: a tip radius of 10 nm, resonance frequency of 87-230 kHz, and force constant of 5 N/m. The number of scan points was 512 or 1024 in each line of the image. The scanning fields were from 100 × 100 *μ*m^2^ to 10 × 10 *μ*m^2^.

### 2.10. Method of Cell Nanosurface Analysis

To analyze the complex structure of a nanosurface and form quantitative evaluation criteria, AFM images of surfaces, using the spatial Fourier transform, were decomposed into two orders with different spectral windows. The first spectral window corresponds to the spatial period *L*_1_ = 600–1000 nm (I order). The second spectral window corresponds to the spatial period *L*_2_ = 50–300 (II order). These spectral windows were chosen so that the surface of each order corresponded to certain structural features of the membrane of RBCs and neutrophils. The I order corresponds to typical membrane parameters. For RBCs, it is flickering, and for neutrophils, it is “wrinkles and folds.” The II order was similar to the size of the cytoskeleton. The heights of structures *h*_1_ and *h*_2_ were not initially specified for each surface; they were measured during the experiments. A more detailed decomposition of the original surface into the sum of surfaces of two or more spatial periods is described in detail in our previous study [44]. On each obtained image of the surface of the first and second orders, the typical parameters of topological nanostructures were measured.

### 2.11. Atomic Force Spectroscopy

The stiffness of the cell membranes was assessed using Young's modulus (*E*). To prepare the sample, the cell sedimentation method was used. Glass with polylysine (MP Biomedicals, France) was used for RBCs.

Cell stiffness was measured using SD-R150-T3L450B cantilevers (Nanosensors, Switzerland) with the manufacturer-provided parameters: a cantilever force constant of 1 N/m and a tip radius of 150 nm. All measurements were performed only on native cells in PBS solution. During the preparation of measurements for each sample, the cantilever was calibrated on glass [45, 46]. To obtain an empirical force curve *I*(*Z*), we set the maximum rise of the piezo scanner *Z*_max_ = 4000 nm and the value of the photodiode current *I* = 0.5 nA. The piezo scanner vertical speed was 0.4-0.8 *μ*m/s. The curves of the stiffness of the samples with neutrophils and RBCs were obtained according to the method described in detail by us earlier [46, 47].

### 2.12. Statistical Analysis

#### 2.12.1. Cells and Membrane Nanostructure

*(1) Analysis of Cell Morphology for Each of the above Exposures*. For each exposure, 3 smears of cell monolayer were prepared. On each smear, 3 areas of 100 × 100 *μ*m^2^ were randomly chosen for AFM scanning. In each area, at least 30 cells were selected for morphological analysis. In total, more than 320 cells were analyzed.

*(2) Analysis of the Nanostructure of Cell Membranes for Each of the above Exposures*. For each of the 3 cell smears obtained for a given impact, there were scanned 5 individual cells selected randomly. For each of these cells, 3 fragments of AFM images of membrane nanostructure (2.5 × 2.5 *μ*m^2^) were analyzed. At each of these fragments, at least 5 measurements of membrane nanostructure characteristics were carried out.

#### 2.12.2. Young's Modulus Measuring

For each type of impact, at least 100 force curves were measured.

#### 2.12.3. Fluorescent Images

To analyze the distance between the actin proteins, 3 samples were prepared for each type of fixative. Airyscan images of RBCs and neutrophils were obtained (*N* = 10 cells from each sample in the amount of 30 cells for each fixative). A total of 90 cells were analyzed. For each image, *n* = 25 segments were measured between the nearest actin proteins, for a total of 750 values for each exposure.

To analyze the colocalization between canals, 3 samples were prepared for each type of fixator. Fluorescent CLSM images of RBCs and neutrophils were obtained (*N* = 10 cells from each sample for a total of 30 cells for each type of fixative). A total of 90 cells were analyzed. The Pearson correlation coefficient and the Manders overlap coefficient were determined.

To assess the significant differences in the data obtained, the Mann–Whitney test was used (^∗^*P* < 0.05,  ^∗∗^*P* < 0.01,  ^∗∗∗^*P* < 0.001, and^∗∗∗∗^*P* < 0.0001). Statistical processing of the results was performed using the program OriginPro 2019 (OriginLab Corporation, USA).

## 3. Results

In the main text of the article, MeOH in concentration 10% and GA in concentrations 0.2% and 0.5% are designated as MeOH10, GA0.2, and GA0.5 correspondingly.

**Figure 2:**
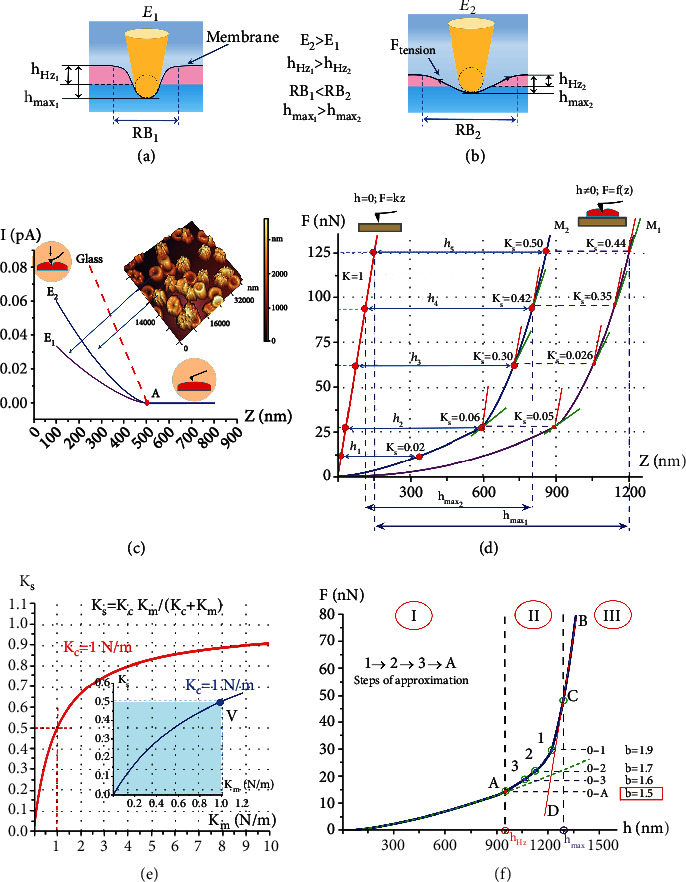
Main biomechanical characteristics of deep bending of blood cell membranes. (a, b) Bending of soft *М*_1_ and hard *М*_2_ membranes. *Е*: Young's modulus; *h*_max_: maximum bending depth; *h*_HZ_: homogeneous bending depth; RB: membrane homogeneous radius. (c) Functions *I*(*Z*) for glass; soft *E*_1_ and hard *E*_2_ membranes. *I* is the photodiode current; *Z* is the distance of the piezo scanner approach; and *A* is the point of contact between the probe and the membrane. (d) Functions *F*(*Z*) for glass (red line); soft *М*_1_ and hard *М*_2_ membranes. *F* is the force acting on the membrane from the side of the probe; *K*_*S*_ is the total force constant. (е) Functions *K*_*S*_ = *f* (*K*_*M*_) for cantilevers with force constant *K*_*C*_ = 1 N/m. (f) Function *F*(*h*).

The AFM probe acts on the membrane with a given force *F*, bending it inside the cell. When the probe is immersed in an elastic membrane, a bending radius RB is formed. If *E*_1_ < *E*_2_, then RB_1_ < RB_2_ (Figures 2(a) and 2(b)). In the AFS method, the empirical curve of the dependence of the photodiode current *I* on the distance *Z* of the piezo scanner feed—*I* (*Z*) is recorded.  Figure 2(c) shows three functions: a straight line—for the interaction of the probe with an absolutely solid body (glass), and curves for two different membranes (*E*_1_ < *E*_2_). The angle of inclination tangent of straight line *I* (*Z*) for the glass is equal to the sensitivity of device. For this case, the following is fulfilled: *F* = *K*_*С*_ *Z* (Hooke's law).

The transition from empirical function *I*(*Z*) (Figure 2(c)) to functions *F*(*Z*) and *F*(*h*) (Figures 2(d) and 2(f)), where *F* is the force acting on the sample and *h* is the indentation depth of the probe:

(1) $$\begin{array}{c} F\left(Z\right)=\frac{I_{m}}{I_{g}}K_{c}Z, \end{array}$$

where *I*_*m*_ *и* *I*_*g*_ are photodiode currents under the action of the indenter on the membrane and glass, respectively, *K*_*C*_ is the force constant of cantilever, and *Z* is the vertical displacement of the piezo scanner.

### 3.1. Biomechanical Characteristics of Deep Bending of the Cell Membrane

When the cantilever was approached to the membrane surface, the point at which the derivative *I*′(*Z*) became more than 0 was the contact point.

The depth of membrane bending *h* is the depth to which the probe is intended under the action of force *F* after touching the membrane surface (point *A*, Figure 2(c)). The value of *h*_*i*_ is determined by the difference in the *Z* coordinates for the glass *Z*_*G*_ and membrane *Z*_*M*_; then, *h*_*i*_ = *Z*_Gi_ − *Z*_Mi_, for line segments *h*_1_–*h*_5_ (Figure 2(d)).

Deep bending of cell membranes is the bending of membranes to a depth of 0.6-0.7 *H* (>1000 nm), where *H* is the height of the native cell on the AFM substrate.

In the mode of membrane deep bending, it is necessary to consider two types of interactions for “cantilever–membrane”: (a) between the tip of the probe and the membrane and (b) between the force constant of cantilever and the force constant of the membrane.

In the resulting Young's modulus of membranes *E*_*S*_ (N/m^2^)—interaction (a), it includes two components connected in series: the Young's modulus of the probe *E*_*C*_ and the Young's modulus of the membrane *E*_*M*_:

(2) $$\begin{array}{c} \frac{1}{E_{s}}=\frac{\left(1-\mu_{c}^{2}\right)}{E_{c}}+\frac{\left(1-\mu_{M}^{2}\right)}{E_{M}}, \end{array}$$

where *μ*_*С*_ and *μ*_*М*_ are the Poisson coefficients for the probe and membrane, respectively. They have numerical values in the range 0–0.5. Equation (2) determines the interaction of the probe material (Si, Ag) with the cell membranes, while, obviously, the condition is always fulfilled: *E*_*C*_ > >*E*_*M*_. This circumstance makes it possible to use the Hertz model to calculate Young's modulus of membranes [46–48]:

(3) $$\begin{array}{c} F=\frac{4}{3}ER^{0.5}h^{1.5}, \end{array}$$

where *E* is Young's modulus, *R* is the radius of the probe tip, and *h* is the depth of membrane bending. It is shown below that Young's modulus for evaluating the mechanical properties of cell membranes can only be used up to the depth of bending *h*_*HZ*_.

In the maximum depth of membrane bending *h*_max_ —interaction (b), in the deep bending mode of membranes, the system for measuring the biomechanical characteristics of membranes consists of two elastic elements connected in series: a cantilever console with a force constant *K*_*C*_ (N/m) (specified by the manufacturer) and a membrane with a force constant *K*_*M*_ (N/m). In this case, the resulting force constant *K*_*S*_ is determined:

(4) $$\begin{array}{c} \frac{1}{K_{S}}=\frac{1}{K_{C}}+\frac{1}{K_{M}}. \end{array}$$

When *K*_*C*_ > >*K*_*M*_ is fulfilled, the force constant of the membrane *K*_*M*_ is recorded (the contribution of *K*_*C*_ is insignificant). If *K*_*C*_ ≈ *K*_*M*_, then the resulting function *F*(*Z*) contains both elastic components: *K*_*C*_ and *K*_*M*_ (Figure 2(e)). At the initial depths of the bending, condition *K*_*C*_ > >*K*_*M*_ is satisfied. As the probe is intended, the stiffness of the membranes increases (as will be shown below), and therefore, the *K*_*C*_ component contribution increases. This circumstance can lead to the fulfillment of condition *K*_*C*_ ≈ *K*_*M*_.

Curves *F*(*Z*) for membranes *M*_1_ and *M*_2_ contain both components: *K*_*M*_ and *K*_*C*_ (Figure 2(d)). On these curves, for several points, the angles between the slope of the straight line for glass *K*_*C*_ (red segments) and the tangents at these points *K*_*S*_ (green segments) are shown, and the values of the resulting force constant *K*_*S*_ at these points are indicated. As the probe is immersed, the tangents *K*_*S*_ approach the slope of the *K*_*C*_ (the angle between *K*_*C*_ and *K*_*S*_⟶min), and the value of *K*_*S*_ increases from 0.02 to 0.4-0.5. At points *К*_*S*_ = 0.4 and higher, the functions *F*(*Z*) are straightened (approximated by a straight line with *R*^2^ at least 0.9) and become close to the slope of the straight line for glass. The value of bending *h*_4_ increases insignificantly, *h*_4_⟶*h*_5_, and ∆h⟶0 in Figure 2(d), or in the general case, *h*_*i*_⟶*h*_*i*+1_. This relationship means that at this depth, the membrane becomes stiff *K*_*M*_ ≈ *K*_*C*_, where *K*_*C*_ = 1 N/m (Figure 2(e)).

In Figure 2(e) (point *V*, blue graph), the measurement area is highlighted in blue. These are the maximum possible areas for measuring *К*_*М*_. If at the initial section of the characteristic *F*(*h*) force constant *K*_*M*_ = 0.02 N/m (Figure 2(f), up to point *A*), then, by the end of the measurement (for *h*_max_, Figure 2(f), after point *C*), the membrane force constant can increase by more than 30 times.

How is point *C* determined? For this determination, from the point of the largest value of the measured *F* (point *B*, Figure 2(f)), the function *F*(*h*) is piecewise approximated by the straight line *F* = *kh* in the direction of decreasing *F*. At the point where the value of *k* changes and the tangent (Figure 2(f), red line *BCD*) breaks away from the curve *F* (*h*), point *C* is localized. In other words, at depths greater than the depth of point *C*, the function *F*(*h*) becomes close to a straight line (approximated by a straight line with *R*^2^ at least 0.9). However, this line is not yet vertical. It can become vertical only when the probe reaches the glass of the substrate; in other words, it pushes the cell through. In the experiment, *h*_max_ reached values of 0.6-0.7 and greater than the height of the cell.

Thus, the maximum membrane bending *h*_max_ (point *С*, Figure 2(f)) is the depth of probe indentation, at which the following conditions are fulfilled: *h*_*i*_⟶*h*_*i*+1_, ∆*h*⟶0, and *К*_*М*_⟶*К*_*C*_.

The depth of homogeneous bending *h*_HZ_ is the depth of probe indentation, up to which the function *F*(*h*) is described by the Hertz model and the condition is fulfilled: *E* = const. The process of probe indentation into the cell membrane is shown in Figures 2(a) and 2(b) and is described by the function *F*(*h*) shown in Figure 2(f).

It is necessary to note that the function *F*(*h*) contains only the force constant of the membrane *К*_*М*_ (there are no components *К*_*С*_ on this function).

This curve can be divided into three sections. Section I (0-A) is described by the Hertz model (Equation (3)) and ends at point *A*, for which the depth of probe indentation corresponds to the value of *h*_HZ_. In this section, the function *F*(*h*) is approximated by the power function *F* = ah^1.5^. Section II (A-1) is the nonlinear part of the function *F*(*h*). The degree of the approximating function *F* = ah^b^ from 0 to any point of section II is not equal to 1.5 (*b* ≠ 1.50 ± 0.02). In the experiment, the degree *b* and the coefficient *a* were fitted by nonlinear fitting of empirical curves. To accomplish this goal, first, an approximating function was selected for the 0-1 section. If *b* ≠ 1.5, then, the approximation procedure was repeated for section 0-2 and so on up to the point for which *b* = 1.50 (Figure 2(f), point *A*). After point *A* (in the direction of increasing *h*), the curve that corresponds to the Hertz model “breaks away” from the empirical curve *F* (*h*) and is shown in the graph (Figure 2(f)) with a green dotted line. In the sections above point 1, the *F*(*h*) dependence is not approximated by a power function, and it is necessary to select polynomials.

Section III of function *F*(*h*) is located at depths *h* > *h*_max_, the boundary of this area, point *C*–depth *h*_max_ (Figure 2(f)).

Thus, in Figure 2(f), section I (0-A) is a zone of homogeneous membrane bending, where the condition *E* = const is satisfied. Section II (A-C) is a section of progressive slowdown, where *E* ≠ const, and *К*_*М*_ is growing. Section III (after point *C*) is a section of linear slowdown, where *K*_*M*_ is maximum. What slows down the probe deepening? As the probe indented, why do the tangents to the function *F*(*Z*) approach the slope of *К*_*C*_, ∆*h*⟶0, and *h* almost stops growing (Figure 2(d))? This outcome is determined by the nonlinear stretching properties of the matrix of RBCs and neutrophils. These phenomena will be discussed below in the model and discussion.

### 3.2. Structural Configuration of Membranes of Nonactivated Neutrophils under the Action of Modifiers

**Figure 3:**
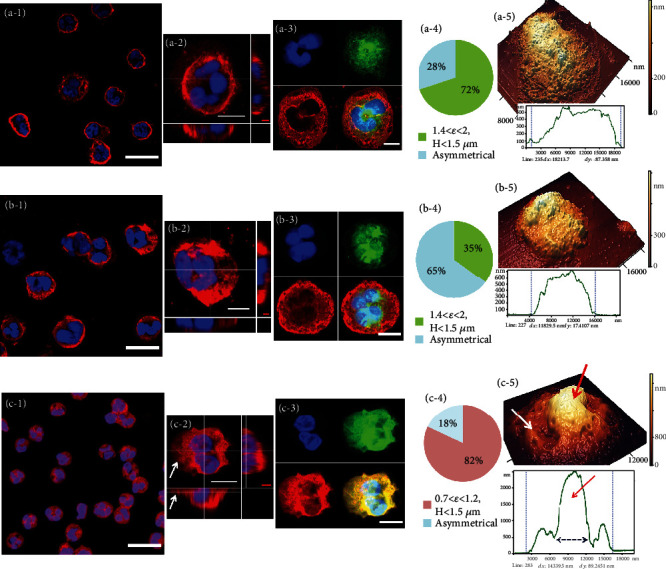
Changes in the structural configuration of neutrophils under the action of modifiers. (a-1) Ensemble of neutrophils by CLSM. (a-2) Single neutrophil by CLSM. (a-3) Individual structural components of the control neutrophil by CLSM. (a-4) Diagram of the distribution of forms of neutrophils in control cells. (a-5) 3D AFM image of a single neutrophil and its profile. (b-1) An ensemble of neutrophils under the action of MeOH10 by CLSM. (b-2) A single neutrophil and its projections along the central axes in the frontal image by CLSM. (b-3) Individual structural components of a neutrophil by CLSM. (b-4) Diagram of the distribution of forms of neutrophils under the action of MeOH10. (b-5) 3D AFM image of a single neutrophil and its profile. (c-1) An ensemble of neutrophils under the action of GA0.5 by CLSM. (c-2) A single neutrophil and its projection (top) by CLSM. (c-3) Individual structural components of a neutrophil by CLSM. (c-4) Diagram of the distribution of forms of neutrophils. (c-5) 3D AFM image of a single neutrophil and its profile; white arrows—membrane halo, red arrows—rise of the central fragment. All CLSM images show calibration marks, and AFM images show colored elevation scales. Color in the field of the CLSM: blue (Hoechst 33342)—DNA nuclei; red (WGA + AF594)—proteins on the membrane (sialic acid and N-acetylglucosaminyl residues), green (phalloidin + AF488)—F-actin. (a-1, b-1, c-1) scale bar = 15 *μ*m; (a-2, b-2, c-2, a-3, b-3, c-3) white scale bar = 5 *μ*m; vertical axis scales for (a-2), (b-2), and (c-2) are shown in red: red scale bar = 1 *μ*m.

**Figure 4:**
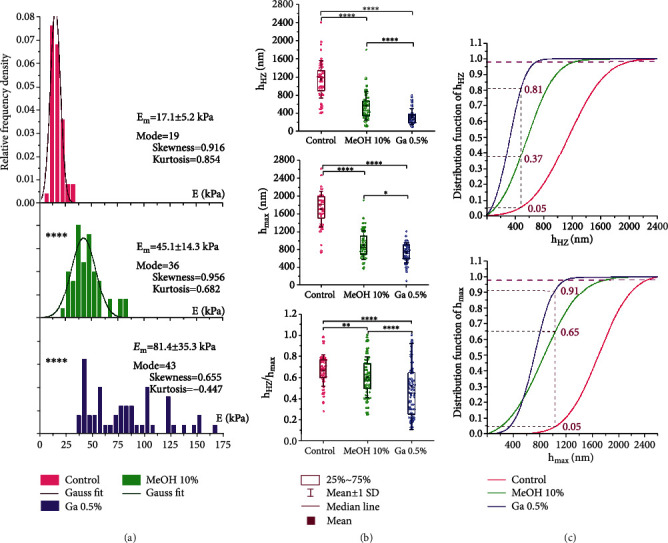
Biomechanical characteristics of membrane deep bending of nonactivated neutrophils under normal conditions and under the action of modifiers: glutaraldehyde—GA0.5; methanol—MeOH10. (a) Histograms of the relative frequencies of Young's moduli *E*; line—approximation by the normal Gaussian law. (b) Changes in the values of *h*_HZ_, *h*_max_, and the ratio *h*_HZ_/*h*_max_. (с) Probability distribution functions for *h*_HZ_ and *h*_max_. Statistics: *n* = 100 for each histogram; ^∗^*P* < 0.05;  ^∗∗^*P* < 0.01;  ^∗∗∗^*P* < 0.001;  ^∗∗∗∗^*P* < 0.0001 (Mann–Whitney test).

**Figure 5:**
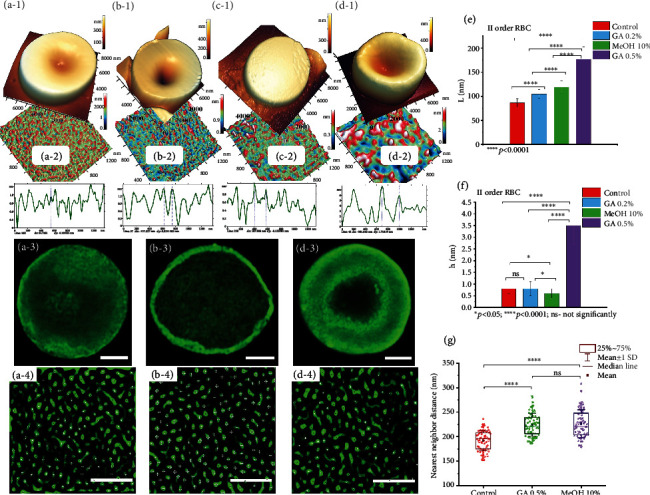
Structural configuration of RBC membranes: in control (a), under the action of GA0.2 (b), МеОН10 (c), and GA0.5 (d). (а-1) 3D AFM image of a single RBC. (а-2) 2D AFM image of the nanostructure of a fragment of the RBC membrane, II order, and its profile; all AFM images show color scales of the heights of the fragments. (а-3) Image F-actin network of RBC and (а-4) its enlarged fragment by СLSM. (b 1–4) The same under the action of GA0.2. (с 1–4) The same under the action of МеОН10. (d 1–4) The same under the action of GA0.5. (e, f) Histograms of the spatial periods and heights of the II order of nanosurface, corresponding. (g) Graphs of changes in distances to the nearest neighbor (nm) for images. F-actin network under modifier actions. Statistics: *n* = 100 for each impact; ^∗^*P* < 0.05;  ^∗∗^*P* < 0.01;  ^∗∗∗^*P* < 0.001;  ^∗∗∗∗^*P* < 0.0001 (Mann–Whitney test). (a-3, b-3, d-3) scale bar = 2 *μ*m; (a-4, b-4, d-4) scale bar = 1 *μ*m.

Upon deposition, the neutrophils acquired the shape shown in Figure 3(a). The average diameter was 10-14 *μ*m, and the size of the nuclear structures was 8-9 *μ*m. The cell components formed the characteristic appearance of the membrane halo (MH) of the neutrophil, shown in red. The most likely type of MH in the control was a symmetric shell with a width of 1-3 *μ*m. The morphology of neutrophils is highly variable, and even *in vitro* at the same temperature and adhesion time, the shapes of the control nonactivated cells could differ from each other. Therefore, for the classification of neutrophils, the following criteria were introduced: *ε* = *S*_*м*h_/*S*_nucleus_is the ratio of the area of MH (S_*м*h_ = S_cell_–S_nucleus_) to the area of the nucleus, and the height of the neutrophil is *H*, which is measured from the AFM image profile (nm) and is the asymmetry of the location of MH relative to nuclear structures. Those neutrophils that were not described by these criteria were assigned to the “others” group. In control neutrophils, 72% of the cells met the criteria: 1.4 < *ε* < 2, *H* < 1.5 *μ*m, and at the same time, they were close to symmetric. Additionally, 28% of the neutrophils were asymmetric (Figure 3(a-4)). The height *H* of the control cells measured by AFM microscopy (Figure 3(a-5)) was 500-1000 nm.

Unstable large “wrinkles” are characteristic membrane formations of neutrophils. To analyze the characteristics of such formations, we used the spatial Fourier transform and the selection of images of nanosurface I and II orders. The spectral window of the first order was 500-1000 nm, which made it possible to isolate large membrane structures. The second order had a spectral window of 50-300 nm and was used to analyze the protein nanolandscape. AFM images of neutrophils and their fragments are shown in Figure 3(series (a), (b), and (c-5)) and in Figure S1. For the control, the characteristic average values of the spatial periods were *L*_1_ = 1879 ± 481 nm, and the average values of the heights were *h*_1_ = 97 ± 35 nm for the first order and *L*_2_ = 361 ± 53 nm and *h*_2_ = 18 ± 6 nm for the second order. In other words, wrinkles appeared on neutrophils at 100 nm or more (Table S1).

The structural configurations of the nuclear complex (blue, 1-sector), membrane proteins (red, 3-sector), actin cytoskeleton (green, 2-sector), and integral staining of all cells (4-sector) are presented in 4 fragments of Figure 3(a-3), Figure S2, and Video S1 and S2. The nuclei had a segmental structure typical of neutrophils. The highest density of the green actin cytoskeleton was formed in the central part of the cell with nuclei, which was confirmed by the colocalization coefficients (Pearson coefficient = 0.868 ± 0.089, *M*_1_ = 0.970 ± 0.044, *M*_2_ = 0.509 ± 0.036) in those areas where dark areas were located in the red image of the membranes—shadows of the nuclear complex (Table S2).

The action of MeOH10 changed the size and shape of the neutrophilic structures (Figure 3(b)). Membrane shells spread, acquired irregular shapes and flattened (Figure 3(b-2)), its projection along the central axes in the frontal image). MHs were located not symmetrically with respect to the nuclear complex and could reach 18–20 *μ*m in diameter. Under the action of MeOH10, such deformed cells accounted for 65% (Figure 3(b-4)). The structural configuration of individual fragments was similar to that of control neutrophils. The maximum density of the actin cytoskeleton (green, sector 2) was located in the central part of the cell, at the location of the nuclear complex, with the following colocalization coefficients: Pearson coefficient = 0.722 ± 0.187, *M*_1_ = 0.984 ± 0.017, and *M*_2_ = 0.333 ± 0.063, and its area was smaller than the total area of membrane structures (red, 3-sector) as in the control (Table S2). The structure of such cells is shown in Figure 3(b-3) and Video S3 and S4. In other cases, the actin cytoskeleton could be located throughout the entire volume of the membrane (Video S5 and S6). However, regardless of the location of the density of the actin complex, the heights of the neutrophils under the action of MeOH10 did not exceed 400-750 nm (Figure 3(b-2), projections along the central axes).

The characteristic changes in the configuration of neutrophils under the action of GA0.5 were different (Figure 3(c)). The central part of the neutrophil membrane, where the nuclear complex was located, was compressed to 5 *μ*m (dashed arrow on the profile Figure 3(c-5)), significantly increasing the height—up to 2-3 *μ*m (Figure 3(c-2) and (c-5)—red arrows on the AFM image and on its profile) (Video S7 and S8). The membrane spread almost symmetrically to 15-18 *μ*m in diameter (Figure 3 (c-2), white arrows). The structural configuration of individual fragments was significantly different from that of control neutrophils (Figure 3(a)). The density configuration of the actin cytoskeleton (green, 2-sector) repeated the configuration of the density of the protein complex of the cell membranes (red, 3-sector); here, the colocalization coefficients were as follows: Pearson coefficient = 0.899 ± 0.063, *M*_1_ = 0.980 ± 0.032, and *M*_2_ = 0.966 ± 0.023 (Table S2). Moreover, its area was comparable to the total area of the membrane structures. In the population, control cells were absent; for 82% of the cells, the criterion was fulfilled: 0.7 < *ε* < 1.2, *H* > 1.5 *μ*m, and the remaining 12% were assigned to the asymmetric group (Figure 3(c-4)).

Under the influence of MeOH10 and GA0.5, the sizes of the nuclear complexes remained almost at the control level. Membrane “wrinkles” increased their heights under the action of MeOH10 by 1.2 times and under the action of GA0.5 by 7.4 times compared with the control in the first order and 2 times and 3.2 times in the second order, respectively (Figure S1). Numerical data on changes in the spatial periods and the heights of the nanosurfaces under the action of modifiers are given in Table S1.

The morphology of neutrophils is determined by the actin cytoskeleton, which stabilizes the cell. To analyze the structural rearrangements of the actin matrix under the action of these modifiers, we recorded the distributions of the centers of the actin network by searching for the nearest neighbors. In the control *l*_*n*,*contr*_ = 251 ± 36 *nm*, under the action of fixators, the components of the cytoskeleton were rearranged, and the actin filaments were shortened: *l*_*n*,*MeOH*10_ = 187 ± 26 *nm* for MeOH10 and *l*_*n*,*GA*0.5_ = 232 ± 37 *nm* for GA0.5 (Figure S3).

### 3.3. Biomechanical Characteristics of Membrane Deep Bending of Nonactivated Neutrophils under the Action of Modifiers

The average value of the neutrophil modulus *E* in the control was 17.1 ± 5.2 kPa. The action of MeOH10 increased this value by 2.6 times, and the addition of GA0.5 increased this value by 4.8 times (Figure 4(a)) (*P* < 0.0001). The statistical distribution of the *E* values in the control was close to the Gaussian law. The stiffer the membranes became (MeOH10, GA0.5), the greater the statistical distribution of *E* differed from the normal law. Thus, in the control, the kurtosis was 0.85; under the action of MeOH10, the kurtosis decreased to 0.68, and with the addition of GA0.5, the kurtosis was already only 0.45. If, under the action of MeOH10, the distribution was asymmetric but still close to normal; then with the addition of GA0.5, a large scatter of *E* values appeared, and the empirical histogram was not approximated by the Gaussian law. This outcome could be due to the appearance of an additional factor of variation—the heterogeneity of the structure of neutrophil membranes and the “patchiness” of the action of the modifier itself. With the introduction of fixators in absolute values, the modes of *E* became less than their average values, which indicated the asymmetry of the distribution toward large values for the *E* values (right asymmetry).

The action of the modifiers caused changes in the biomechanical characteristics of deep bending of neutrophil membranes. The value of *h*_max_ under the action of MeOH10 decreased 1.4 times compared with the control and, under the action of GA0.5, decreased 1.7 times. The *h*_*HZ*_ parameter also had a tendency toward a decrease in the absolute values under the action of these modifiers. In the control, the average value of *h*_*HZ*_ was 1147 ± 413 *nm*; under the action of MeOH10, this value decreased by 2 times, and under the action of GA0.5, it decreased by 3.5 times. Numerical values for the absolute values of *h*_max_ and *h*_*HZ*_ in the control and under the action of MeOH10 and GA0.5 are given in Table S1.

It is important here that the decrease in *h*_HZ_ proceeded faster than *h*_max_. Rearrangements in the structure of membranes and matrix under the action of Me and GA0.5 caused various effects of changes in the deformation parameters of membrane deep bending. The absolute values of the *h*_HZ_ values depended on the maximum possible bending of the membranes. Obviously, the greater the depth bending, the greater the absolute value of *h*_HZ_ should be. However, the relative changes in *h*_HZ_/*h*_max_ had a statistically significant tendency to decrease with an increase in the Young's modulus of the membranes. In the series of control–MeOH10–GA0.5, the Young's modulus of membranes increased to 17.1–45.1–81.4 kPa, and the fraction of the *h*_HZ_ value in relation to *h*_max_ decreased: 0.67–0.63–0.45 (Table S1), respectively. In other words, an increase in the stiffness of neutrophil membranes was always accompanied by a decrease in the depth of linear uniform bending of their membranes. MeOH10 had little effect on shortening the depth of the *h*_HZ_, but GA0.5 reduced this parameter by almost 1.5 times (Figure 3(b)).

The histograms of the relative frequencies for *h*_HZ_ and *h*_max_ were approximated by the normal Gaussian distribution *f*(*h*_HZ_) and *f*(*h*_max_). To analyze the changes in the values of *h*_HZ_ and *h*_max_ under the action of neutrophils MeOH10 and GA0.5 on the membranes, the corresponding probability distribution functions *F*_*d*_(*h*_HZ_) and *F*_*d*_(*h*_max_) were constructed:

(5) $$\begin{array}{c} F_{d}\left(h_{\text{HZ}}\right)=\int_{-\infty }^{h_{\text{HZ}}}f\left(h_{\text{HZ}}\right)dh_{\text{HZ}},\\ F_{d}\left(h_{\max}\right)=\int_{-\infty }^{h_{\max}}f\left(h_{\max}\right)dh_{\max}. \end{array}$$

On the graphs of the functions in Equation (5), a level of 0.05 was plotted, which corresponded to *h*_max_ = 1040 nm for the control and *h*_HZ_ = 470 nm. At these levels, probability distribution functions were used to determine what percentage of cells for MeOH10 and GA0.5 retained their values compared to the control values (Figure 4(c)). Thus, under the action of MeOH10, such cells remained 35% for *h*_max_ and 63% for *h*_HZ_. Under the action of GA0.5, there were significantly fewer cells: only 9% for *h*_max_ and 19% for *h*_HZ_. The numerical values of the proportions of cells differing from the control are given in Table S1.

### 3.4. Structural Configuration of Red Blood Cell Membranes under the Action of Modifiers

Figure 5 series 1 (horizontal) shows the characteristic forms of RBCs, which under the action of this modifier, accounted for more than 60% of the cells. In the control, these are classic discocytes (Figure 5(a)); GA0.2 transformed it into a cell with a deep cavity (Figure 5(b)). Under the action of MeOH10, planocytes appeared (Figure 5(c)), and GA0.5 sharply increased the height of the cell while decreasing its size (Figure 5(d)).

Nanosurfaces of the second order of membranes are of particular interest (Figure 5 series 2) since they reflect the structural configuration of the surface protein landscape in control and under the influence of modifiers [49]. The modifiers increased the average spatial period *L*_2_ in comparison with the control values: for GA0.2 and MeOH10 it was almost the same—1.4 times greater, and for GA0.5, it was 2 times greater. The mean values of the local heights *h*_2_ for the control, GA0.2, and MeOH10 were statistically indistinguishable. However, GA0.5 sharply changed the parameters of the membrane nanosurface: the average value of period *L*_2_ increased by 2.0, and the average value of height *h*_2_ increased by 4.4 (Figures 5(e) and 5(f)). These effects are quantitatively illustrated in the respective profiles (Figure 5, series 2) and in Table S1. In other words, the effect of GA0.5 on the structure of the membrane nanosurface was the most pronounced in comparison with other modifiers.

Structural rearrangements of the RBC cytoskeleton under the action of modifiers were analyzed by the distribution of F-actin centers using nearest neighbor search programs (Figure 5, sectors 3 and 4). In the control, the value *l*_RBC,contr_ = 192 ± 20 nm. This value corresponded to the distance between the junctions of the cytoskeleton—the size of the double spectrin filament (single strand length ≈ 100 nm), which indicated the normal state of the RBC cytoskeleton network. Under the action of MeOH10 and Ga0.5, the *l*_RBC_ value increased almost equally, up to *l*_RBC,MeOH10_ = 226 ± 24 nm and up to *l*_RBC,GA0.5_ = 224 ± 23 nm (Figure 5(g)). The modifiers caused complex rearrangements of the cytoskeleton and membrane nanostructure, leading to the effect of cell fixation. These realignments were interdependent [18, 50, 51].

### 3.5. Biomechanical Characteristics of Deep Bending of RBC Membranes under the Action of Modifiers

**Figure 6:**
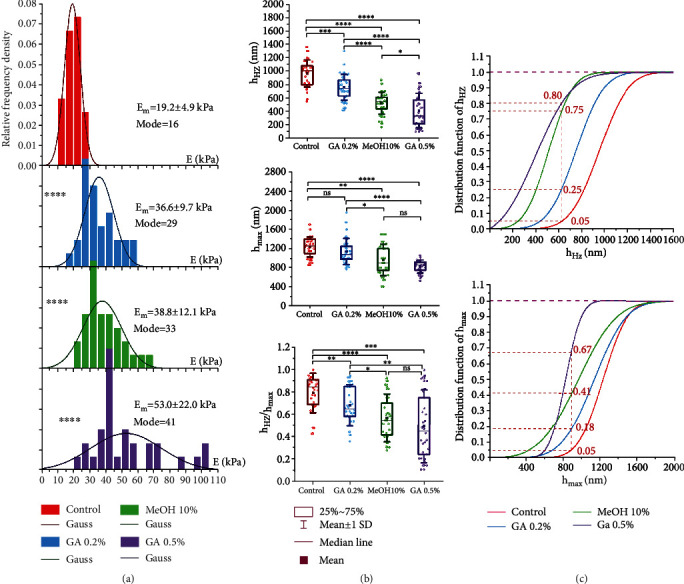
Biomechanical characteristics of RBC membranes under normal conditions and under the action of modifiers: GA0.2, MeOH10, and GA0.5. (а) Histograms of the density of relative frequencies of Young's moduli *E*; lines—Gaussian normal law. (b) Changes in the values of *h*_HZ_ and *h*_max_. (с) Probability distribution functions for *h*_HZ_ and *h*_max_. Statistics: *n* = 100 for each histogram; ^∗^*P* < 0.05;  ^∗∗^*P* < 0.01;  ^∗∗∗^*P* < 0.001;  ^∗∗∗∗^*P* < 0.0001 (Mann–Whitney test).

Young's modulus *E* of control native RBCs had an average value of 19.2 ± 4.9 kPa, and its distribution was close to the normal Gaussian law. The addition of GA0.2 and MeOH10 increased the module almost equally: by 1.9 and 2.0 times, respectively. GA0.5 had the greatest effect—an increase of 2.7 times (Figure 6(а) and Table S1). As the average value of Young's modulus increased, the histograms shifted toward larger values, and at the same time, the variance increased. The modes in all cases were less than the mean, which indicated a right-handed asymmetry in the distribution. The empirical histograms of *E* modules under the action of all modifiers were adequately described by the normal law, and under the action of GA0.5, multimodal distributions did not appear, as in neutrophils. The reason is that the structural configuration of RBCs is simpler and more stable than that of neutrophils.

Trends in the *h*_HZ_ and *h*_max_ values were similar: as Young's modulus increased, these values decreased (Figure 6(b)). In other words, the stiffer the membrane became, the less depth it could bend (evident effect). However, these changes were not proportional. Here, a nonlinear effect of the deformation properties of the membrane structure arose: the greater the value of Young's modulus was, the smaller the ratio of the homogeneous bending (*E* = const) to the maximum possible bending (*h*_HZ_/*h*_max_) (Table S1). Thus, in the control–GA0.2–MeOH10–GA0.5 series, the proportion of zone of the homogeneous bending (*h*_HZ_/*h*_max_) decreased by 0.79–0.68–0.56–0.49.

With changes in the values of Young's modulus, some of the RBCs retained the properties of the control (Figures 6(a) and 6(c)). With the growth of *E*, there were fewer such cells. To assess the proportion of such cells on the graphs of functions (Equation (5)), the level of 0.05 was plotted, which corresponded to *h*_max_ = 640 nm for the control and *h*_HZ_ = 890 nm. At these levels, probability distribution functions were used to determine what percentage of cells for MeOH10 and GA0.5 retained their values to be the control values (Figure 6(c)). Thus, under the action of GA0.2, 80% of these cells remained for *h*_max_ and 75% remained for *h*_HZ_. Under the action of GA0.5, such cells remained significantly less: 33% for *h*_max_ and 20% for *h*_HZ_. The numerical values of the proportions of cells differing from the control are given in Table S1.

### 3.6. The Nature of the Nonlinear Deformation of Cell Membranes: Mathematical Model

In this section, we discuss the nature of inhibition that occurs during the probe indentation into the membrane during its deep bending. We attempt to answer the question of why, during deep bending, the slope of the function *F*(*h*) for membranes approaches that for glass and to consider the reasons for the appearance of the characteristic parameters *h*_max_ and *h*_HZ_.

**Figure 7:**
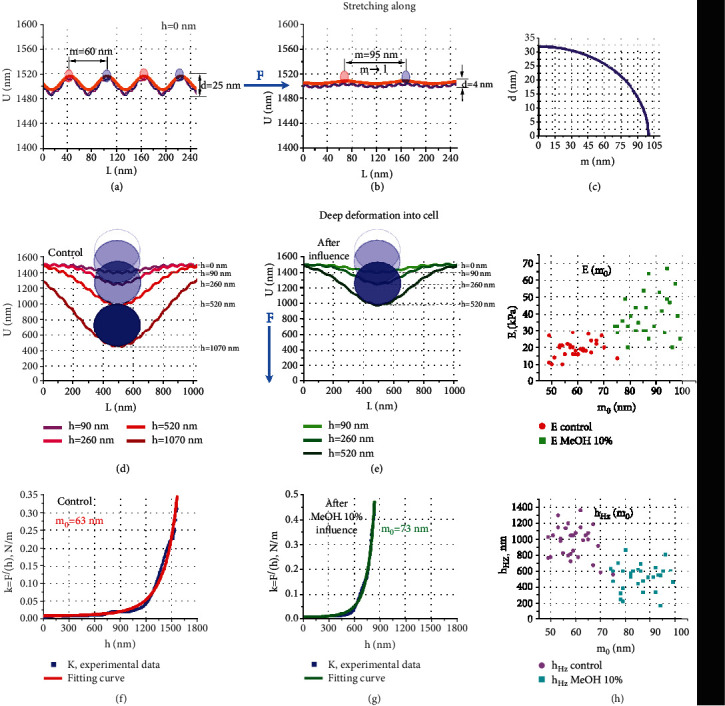
Mathematical model of nonlinear deformation of membranes. (a, b) Two-dimensional fragment of the cytoskeleton mesh in the plane of the surface of RBCs in the initial state (a) and in the state stretching along (method a) (b). (c) Dependence of the helix diameter *d* of the spring on the helix pitch *m* at a constant contour length *l*. (d, e) Fragment of the cytoskeleton mesh in a plane perpendicular to the surface of RBCs, with bending of the membrane into the cell (method b) in control (d) and under the action of the MeOH10. (f, g) Experimental data *k*(*h*)_exper_ = *F*^/^(*h*) (blue lines) for control (f) and after exposure to MeOH10 (g). Nonlinear curves fitting experimental data according to Equation (10) for control cells (f) (red) and after exposure to MeOH10 (g) (green). (h) Experimental dependences of Young's modulus *E* and bending depth *h*_Hz_ of the membrane (spring) on the initial helix pitch *m*_0_, calculated in the model. Red and lilac—control; green and blue—after exposure to MeOH10.

The mechanical state plays a key role in the function of blood cells. Mechanical properties are determined by the state of the structural elements of the cell [49]. The main structural element of RBCs is the cytoskeleton, which is a volumetric network formed on the basis of spectrin filaments connected to the lipid bilayer by a complex of proteins [52, 53]. Figures 7(a) and 7(b) are schematic representations of a two-dimensional network fragment in a plane perpendicular to the surface. The main junctions of attachment of the cytoskeleton to the membrane are actin (red circle) and ankyrin (blue circle) complexes (Figures 7(a) and 7(b)). It is the cytoskeleton network that allows RBCs to change their shape and thereby circulate in the capillary system. The mechanism of stiffness of the cytoskeletal RBC network continues to be discussed in the scientific literature [2, 49, 52–54].

The RBC cytoskeleton is a single three-dimensional elastic filament structure with entropic elasticity [55]. The altered configuration of individual filaments in a local area leads to a change in neighboring structures and, in general, in the collective elasticity of the entire cytoskeleton. The characteristic sizes of the cytoskeletal elements are determined by the size and mechanical properties of the spectrin filaments, the protein complexes at the junction nodes, the state of the lipid layer, and the osmotic pressure in RBCs. All of these parameters can change as a result of the action of physicochemical factors on blood cells (in the work, MeOH10, GA0.2, and GA0.5). Changes in the parameters of the cytoskeleton are the causes of changes in the nanostructure, membrane stiffness, and morphology of RBCs. These changes can reduce the deformability of cells and disrupt their functions [2].

The problems of membrane large deformation modeling are discussed in the scientific literature. An example of effective modeling of deformations in a spectrin network is the model described in the article [56].

Our model assumes the following assumptions: cytoskeletal filaments are considered to be helical springs; and windings are formed by spectrin dimers, which do not change their length and properties up to their stretching of 95–97 nm.

Let us consider the basic equations that connect the helix parameters [57, 58]. Based on the equations presented in study [57], it follows that the helix diameter *d* (the bending depth/filament sagging) is related to helix pitch *m* and contour length *l* (Figure 7(c)):

(6) $$\begin{array}{c} d\left(m\right)=\frac{1}{\pi }\sqrt{\;\left(l^{2}-m^{2}\right)}. \end{array}$$

Analysis of curve *d*(*m*) shows that there are two special regions: in the first, *m* = 30–40 nm, in which the first derivative *d*^/^(*m*) begins to increase, and in the second, *m* = 70–80 nm, in which the second derivative *d*^//^(*m*) begins to increase. In the first area, the size *d* of the sagging of the cytoskeleton elements almost does not change with an increase in the distance between the junctions. In the second region, at already strong stretching of the filaments, *m* > 75 nm, a sharp effect of *m* on *d* occurs.

The spring constant depends on the diameter of the helix, as follows [58]:

(7) $$\begin{array}{c} k=\frac{A}{d^{3}}. \end{array}$$

By increasing the helix pitch *m*, its diameter will decrease according to Equation (6), and the spring constant *k* will increase nonlinearly according to Equation (7).

There are two ways to stretch the cytoskeleton spring:

- Apply a force in the horizontal direction in the model in (Figures 7(a) and 7(b)),
- Bend the membrane in a direction perpendicular to the surface (Figures 7(d) and 7(e)), for example, by the probe in the AFS method or under blood pressure in microvessels.

Let us analyze the increase in the spring constant of a helix formed by spectrin dimer when it is stretched under the action of a horizontally applied force (method a). Based on the data on the dependence of the force on the stretching length of spectrin filaments [59], we found that spring constant *k*(*m*)_exper_ = *F*^/^(*m*)_exper_. For nonlinear curve fitting experimental data, we used a function of the form

(8) $$\begin{array}{c} k\left(m\right)=\frac{a}{\;{\left(l^{2}-m^{2}\right)}^{3/2}}, \end{array}$$

There were established unknown parameters of the approximation *a* and *l* (*R*^2^ = 0.99); this indicates the adequacy of the model (Equation (8)). The value *l* = 96.7 nm corresponds to an almost complete stretching of the spectrin filament; *a* = *π*^3^*A*.

To solve the problem of deep bending of the membrane (method b, Figures 1(d) and 1(e)), it is necessary to know how the helix pitch *m* changes depending on the bending depth of the membrane *h*, *m*(*h*). The most adequate approximation of the increase in the helix pitch with deep bending of the membrane is the function

(9) $$\begin{array}{c} m\left(h\right)=l\left(1-\frac{m_{0}}{l}\right)\int_{-\infty }^{h}\frac{1}{\sqrt{2\pi }d}e^{-\frac{{\left(h-c\right)}^{2}}{2d^{2}}}dh+m_{0}. \end{array}$$

Different values *m*_0_ take place in reality, as evidenced by 3D AFM images of fragments of the RBC membrane nanosurface 1300 × 1300 nm^2^ control and after exposure to MeOH10, as well as the corresponding height profiles (Figure 5(a-c) and (d-2). The histograms (Figure 6) show that after exposure to MeOH10, the initial distance *m*_0_ between the actin and ankyrin complexes on average increases compared to the control and the height of the nanostructure (corresponding to the helix diameter) decreases, which is in good agreement with Equation (6).

With increasing membrane bending *h*, membrane spring constant *k* will increase due to an increase in *m* and a corresponding decrease in *d* (Figure 7(c)). Let us apply the model (Equations (8) and (9)) to approximate the experimental data *k*(*h*)_exper_ = *F*^/^_exper_(*h*) when the membrane is bent by the probe (Figures 7(f) and 7(g)):

(10) $$\begin{array}{c} k\left(h\right)=k\left(m\left(h\right)\right)=\frac{a\;}{{\left(l^{2}-m^{2}\left(h\right)\right)}^{3/2}},\\ k{\left(h\right)}_{\text{theor}}=\frac{a}{{\left[l^{2}-{\left(l\left(1-\left(m_{0}/l\right)\right)\int_{-\infty }^{h}\left(1/\sqrt{2\pi }d\right)e^{-{\left(h-c\right)}^{2}/2d^{2}}dh+m_{0}\right)}^{2}\right]}^{3/2}}. \end{array}$$

Unknown approximation coefficients *a*, *m*_0_, *c*, and *d* are selected in the nonlinear fitting method in such a way that the experimental data *k*(*h*)_exper_ and theoretical data *k*(*h*)_theor_ with *R*^2^ ≥ 0.95 coincide in the best way.  Figured 7(f) and 7(g) (blue) present the experimental data *k*(*h*)_exper_ = *F*^/^(*h*)_exper_ obtained from plots *F*(*h*) (Figure 2(f)). According to Equation (10), fitting of the control data (red) was conducted, and it was obtained for membranes of control cells in the examples shown in Figure 7(f): *m*_0contr1_ = 63 nm, *m*_0contr2_ = 50 nm, and *m*_0contr3_ = 62 nm. According to Equation (10), fitting the data was conducted after exposure to MeOH10 (green), and it was obtained for the cell membranes in the examples presented in Figure 7(g): *m*_0MeOH1_ = 73 nm, *m*_0MeOH2_ = 89 nm, and *m*_0MeOH3_ = 87 nm.

Nonlinear curve fitting (by Equation (10)) of our experimental data made it possible to estimate the initial parameters of the membrane nanostructure, namely, the initial distances between the actin and ankyrin complexes (the initial helix pitch): in the control *m*_0contr_ = 59 ± 6 nm, after exposure to MeOH10 *m*_0MeOH10_ = 86 ± 8 nm. Figure 7(h) presents data on the control and MeOH10. For the effect of GA0.5, it was found that *m*_0GA0.5_ = 90 ± 8 nm. These data correlate well with the data obtained on the basis of AFM studies of the parameters of the nanosurface of RBC membranes.

Figure 7(h) presents graphs that show the experimental data for Young's modulus *E*(*m*_0_) and bending depth *h*_Hz_(*m*_0_), corresponding to the values of the initial helix pitch *m*_0_, calculated in the model. Numerical data for the control are highlighted in red and lilac and, after exposure to MeOH10, in green and blue. With increasing *m*_0_, there is a tendency for an increase in *E* and a decrease in *h*_Hz_. The Pearson correlation coefficients were  *r*_*E*/*m*_0__ = 0.726 and *r*_*h*_*Hz*_/*m*_0__ = −0.728.

In the future, the presented model can be developed to assess the nanoparameters of the cytoskeleton of neutrophils on the basis of AFS to probe deep bending of their membranes.

The presented mathematical model Equation (10) allows solving the inverse problem: quantitatively assessing the nanoparameters of the cytoskeleton using the measured force curves. The model shows that an increase in the initial distance between the actin and ankyrin complexes can be the cause of an increase in Young's modulus *E* and a decrease in the homogeneous bending depth of the membrane *h*_Hz_. These parameters can be used as diagnostic indicators to quantify the degree of various effects on cell membranes.

## 4. Discussion

In neutrophils, these fixatives changed the shape and size of MH, the height of the cell, the parameters of the nanosurface of orders I and II, and the location and size of the actin cytoskeleton. At the same time, in RBCs, one changed the morphology, nanosurface parameters of orders I and II, and the nearest neighbor distance of the cytoskeleton actin network.

### 4.1. Deformation Capacity of Membranes

The deformation capacity of the membranes of neutrophils and RBCs was described by the following parameters: Young's modulus *E*, spring constant of membranes *К*_*М*_, depth of homogeneous bending *h*_HZ_, depth of maximum bending *h*_max_, and the proportion of depth of homogeneous bending to the maximum: *h*_HZ_/*h*_max_.

**Figure 8:**
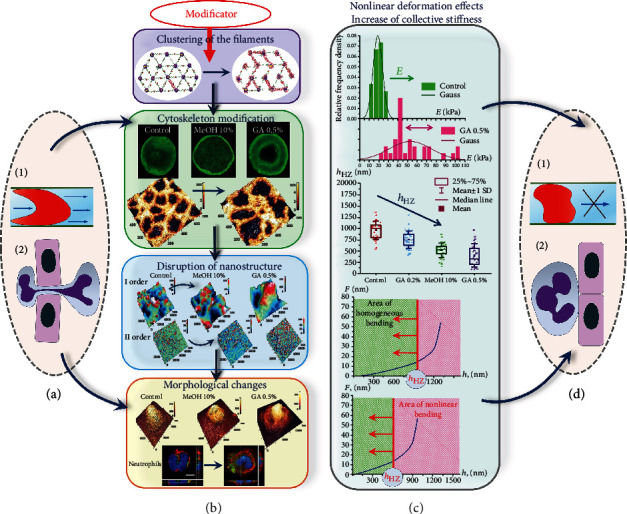
The structural configuration of neutrophils and RBCs determines the nonlinear deformation properties of their membranes during deep bending. (a) The biomechanical characteristics of the membranes of RBCs and neutrophils ensure their mobility in the capillary network and in tissues. (b) Action of modifiers: vertically from top to bottom: clustering of cytoskeleton filaments, rearrangements of cytoskeleton configuration, and changes in the membrane nanosurface and morphology. (c) The result of the action of modifiers—an increase in the collective stiffness and the occurrence of nonlinear deformations; vertically from top to bottom: an increase in Young's modulus causes its multimodal distribution, a decrease in the *h*_HZ_ parameter, and a reduction in the zone of homogeneous bending. (d) As a result of changes in the biomechanical characteristics of the membranes of RBCs and neutrophils, their mobility in the capillary network and in tissues is impaired.

All modifiers significantly increased Young's modulus of membranes (up to *h*_HZ_ depth) in neutrophils and RBCs (Figures 4 and 6). The average value of *E* became greater (in the control, the increase in neutrophils was 4.8 times and, in RBCs, 2.7 times), and at the same time, the distribution variance increased and the value *h*_HZ_ became lower by 3.5 and 2.3 times, respectively (Figures 4 and 6, Table S1). The correlation coefficient between the average values of *E* and *h*_HZ_ reached -0.9 with the action of all modifiers. In all experiments, for both neutrophils and RBCs, an increase in the stiffness was accompanied by a reduction in the zone of homogeneous bending of the membrane (Figures 4(b), 6(b), and 8(c)) and an increase in the number of cells with large values of *E* (right distribution asymmetry). At the same time, there was fragmentation (spotting, clustering) of the action of fixators (Figure 6(a)), especially manifested under the action of GA0.5 on neutrophil membranes (Figure 4(a)).

The process of deep deformation of membranes was divided into three zones. The zone of homogeneous bending (zone I, Figure 2(f)) is studied and widely discussed in all work devoted to this topic [17, 33, 37, 46, 47]. The zone of progressive inhibition (zone II) in Figure 2(f) is shown from point *A* to point *C*. Point *A* is specified by the value of *h*_HZ_ definitely and precisely. The same cannot be said about the position of point *C*—the value of *h*_max_. Point *C*, strictly speaking, is conventionally defined as the point of separation of the line *B*–*C*–*D* on the function *F*(*h*) (Figure 2(f)). However, in favor of such a representation, the following considerations can be stated. The spring constant in section III remain close in the control and under the action of all used modifiers—in a narrow range of 0.1—0.3 N/m. The *h*_max_ parameter is distributed according to the Gaussian law for all influences. If the *h*_max_ parameter changes only in a narrow range, then, this change could mean that it is a function of the same physical phenomenon (discussed below).

In this regard, it is interesting to note that the force constant *К*_*М*_ for control cells from site I to site III increased 10-15 times, and for GA0.5, it increased only 4-5 times. The reason was that the *К*_*М*_ in the initial section I for GA0.5 was already 3-4 times higher than that in the control. Since in the final section, section III, the processes are determined by the same phenomenon, the final values of the *К*_*М*_ should be close, and therefore, the increase in elasticity for GA0.5 should have been less. Since the stiffness of section III for all actions was determined by one argument, such a parameter can be adequately used in measurements.

### 4.2. The Cause of Nonlinear Effects at Deep Bending of Membrane

There is a fundamental difference between the elastic modulus and the stiffness of the membrane. The elastic modulus is a property of an isotropic material, and the mechanical stiffness (spring constant) *К*_*М*_ is a property of a structure or its components. The membrane is not a homogeneous and not isotropic. It is a *complex* structure: a lipid bilayer connected to a network of spectrin cytoskeleton by protein complexes in RBCs and an actin network connected to the microtubules of the cytoskeleton and the surface layer of the membranes in neutrophils. The biomechanical characteristics of such structures depend not only on the intrinsic properties of the structures themselves but also on the value of their deformations (bending).

Hertz's model takes into account an increase of force due to an increase in the probe-membrane contact area and, consequently, a change of effective force constant. But, as shown in our experiments, at depths larger than *h*_HZ_, the membrane force constant increases faster than isotropic homogeneous deformation suggests.

An increasing number of fragments of membrane structure enter into deformation processes. At *h* < *h*_HZ_, there was a homogeneous bending. Then, gradually, the cytoskeleton network entered the process, and membrane stiffness increased (Figure 2(f), zone II). When the filaments of the cytoskeleton straighten and lose winding elasticity, the collective springiness of the cytoskeleton network comes into play (Figure 7). If the spring of the cytoskeleton is still stretched in section II, then in section III, the cytoskeleton network is already fully stretched (Figure 2(f)). As a result, the cytoskeleton bends into the cell in a large fragment. Therefore, curve *F*(*h*) in section III is close to linear: it is approximated by the direct function *F* = *K*_*M* (III)_ *h*, with *R*^2^ being not less than 0.9. At the same time, the lateral tension forces *F*_tension_ of the entire membrane increase (Figure 2(b)).

### 4.3. Measurement Tools

To measure the biomechanical characteristics of deep bending, stiff cantilevers are used (in our work, *К*_*С*_ = 1 N/m) such that the slope of the *F*(*Z*) curve (Figure 2(d)) becomes close to the angle of inclination of the straight line for glass (in other words, close to *К*_*С*_, as in Figures 2(e) and 2(f)) at bending depths of 0.6-0.7 of the cell height *H* (*h*_max_ is approximately 1200-1300 nm and more). The viscoelastic properties of membranes under conditions of large deformations (deep bending) can be described by constant Young's modulus *E* only up to the depth *h*_HZ_. At the bending of membranes to depths larger *h*_HZ_, the deformation process can be correctly described by the variable force constant of the membranes *К*_*М*_ (*h*) = *dF*/*dh* (Equation (10)). When using soft cantilevers (for example, *К*_*С*_ = 0.05 N/m), the probe does not reach the depths that correspond to point *A* and, moreover, point *C* (Figure 2(f)). Soft cantilevers are adequately used for small probe indentation (0.1–0.3 *H*).

### 4.4. Results and Consequences

Figure 8 shows the progress and the results of the work as a whole. The rearrangement of the structural configuration of the membranes of neutrophils and RBCs caused nonlinear deformation phenomena that could lead to disturbances in the rheological properties of blood.

In fragment A, neutrophils and RBCs are well deformable and therefore migrate through tissue gaps and the capillary system. In fragment B, modifiers act on the structure of cells, rebuilding it. These rearrangements are caused by breaks and clustering of filaments of the cytoskeleton, changes in the membrane nanosurface, and morphology of neutrophils and RBCs. In fragment C, there is an increase in the collective stiffness and the emergence of nonlinear deformation effects: there are an increase in *E* and its dispersion, a decrease in the parameter *h*_*HZ*_, and a reduction in the zone of homogeneous bending of the membrane. In fragment D, the consequences of the processes shown in fragment C disrupt the functionality of neutrophils and RBCs: penetration into the capillary system and tissue gaps is difficult.

### 4.5. Problems

In the process of performing the work, problems were identified that require further research.

Despite the above considerations, the problem of interpreting section III and point *C* remains. This problem is a separate problem that requires further research and discussion.

How does the deformation occur after the depth *h*_max_? What happens to the membrane at depths on the order of 0.8-0.9 of the cell height? The answer to these questions also requires separate research.

The membrane bends in the zone of the probe and stretches over the entire surface, since the volume of the cytoplasm does not change. Apparently, lateral tension forces *F* can affect the deformation processes and biomechanical characteristics of membranes. What is the possible contribution of these forces to the overall deformation process? This problem requires separate consideration.

## 5. Conclusion

In this work, we linked changes in the structure of the membranes of neutrophils and RBCs under the action of cytological fixatives with the occurrence of nonlinear deformation effects of deep bending of these cells. The results of this work can be useful in studying the deformation properties of blood cell membranes under normal conditions and in various diseases. These results can also be used in clinical practice in the treatment of blood pathologies and in the development of new drugs.

## Data Availability

The datasets used and analyzed during the current study are available from the corresponding authors on request.

## Supplementary Materials

Supplementary Materials Figure S1: AFM images of typical nanosurfaces of orders I and II of whole neutrophils and under the action of MeOH10 and GA0.5. AFM images of typical nanosurfaces of orders I and II of whole neutrophils and their regions and the corresponding nanosurface profiles of the control neutrophil region (without any exposure) when exposed to MeOH10 and when exposed to GA0.5. The bottom row shows histograms of the spatial periods *L* and heights *h* of the first and second orders. Statistics—*n*: 150 profiles from 5 cells; ^∗^*P* < 0.05;  ^∗∗^*P* < 0.01;  ^∗∗∗^*P* < 0.001;  ^∗∗∗∗^*P* < 0.0001; ns: not significantly (Mann–Whitney test). Figure S2: imaging of dried neutrophil by CLSM. (a) Neutrophil, dried at room temperature in air. (b) Lateral projection of neutrophils dried at room temperature in air. Image captured using Zeiss Airyscan module. Channels: blue (Hoechst33342)—DNA, green (phalloidin + A488)—F-actin, and red (WGA + A594)—membrane. The image shows the spatial position of the nuclei, membrane, and F-actin. The difference is seen in the position of the membrane and F-actin. F-actin occupies a smaller portion of the membrane area and is colocalized with the nuclei. Scale bar = 5 *μ*m. Figure S3: evaluation of the parameters of the nanostructure of the actin cytoskeleton of neutrophils. (a) Maxima of neutrophil actin fluorescence. 1—neutrophil dried at room temperature in air, 2—neutrophil fixation with GA0.5, and 3—neutrophil fixation with MeOH10. The top image is the whole cell. Scale bar = 5 *μ*m. The bottom image is a 3 × 3 *μ*m^2^ region with marked maxima of actin fluorescence. Scale bar = 1 *μ*m. (b) The nearest neighbor distance analysis for F-actin in neutrophils. *N* = 3 cells. 25 points from each cell. ^∗∗^*P* < 0.01;  ^∗∗∗∗^*P* < 0.0001 (Mann—Whitney test). (c) A table of the nearest neighbor distance values; mean ± SD. Table S1: biomechanical parameters and characteristics of the nanostructure of neutrophils and RBC under the action of modifiers. Table S2: the colocalization coefficients for neutrophils under the action of modifiers. Video S1: animated Z-stack of neutrophils was dried at room temperature in air. Blue (Hoechst33342)—DNA. Green (phalloidin + A488)—F-actin. Red (WGA + A594)—membrane. Scale bar = 5 *μ*m. Video S2: animated lateral neutrophil projections were dried at room temperature in air. Blue (Hoechst33342)—DNA. Green (phalloidin + A488)—F-actin. Red (WGA + A594)—membrane. Scale bar = 5 *μ*m. Video S3: animated Z-stack of neutrophil, fixed with MeOH10. Blue (Hoechst33342)—DNA. Green (phalloidin + A488)—F-actin. Red (WGA + A594)—membrane. Scale bar = 5 *μ*m. Video S4: animated lateral neutrophil projection, fixed with MeOH10. Blue (Hoechst33342)—DNA. Green (phalloidin + A488)—F-actin. Red (WGA + A594)—membrane. Scale bar = 5 *μ*m. Video S5: animated Z-stack of neutrophil, fixed with MeOH10. Blue (Hoechst33342)—DNA. Green (phalloidin + A488)—F-actin. Red (WGA + A594)—membrane. Scale bar = 5 *μ*m. Video S6: animated lateral neutrophil projection, fixed with MeOH10. Blue (Hoechst33342)—DNA. Green (phalloidin + A488)—F-actin. Red (WGA + A594)—membrane. Scale bar = 5 *μ*m. Video S7: animated Z-stack of neutrophil, fixed with GA0.5. Blue (Hoechst33342)—DNA. Green (phalloidin + A488)—F-actin. Red (WGA + A594)—membrane. Scale bar = 5 *μ*m. Video S8: animated lateral neutrophil projection, fixed with GA0.5. Blue (Hoechst33342)—DNA. Green (phalloidin + A488)—F-actin. Red (WGA + A594)—membrane. Scale bar = 5 *μ*m.
