# Supplementary material for: Structural Configuration of Blood Cell Membranes Determines Their Nonlinear Deformation Properties
Source: Biomed Res Int. 2022 Apr 18;2022:1140176. doi: 10.1155/2022/1140176 (PMC9038403; doi:10.1155/2022/1140176)
Supplement: Supplementary Materials — Figure S1: AFM images of typical nanosurfaces of orders I and II of whole neutrophils and under the action of MeOH10 and GA0.5. AFM images of typical nanosurfaces of orders I and II of whole neutrophils and their regions and the corresponding nanosurface profiles of the control neutrophil region (without any exposure) when exposed to MeOH10 and when exposed to GA0.5. The bottom row shows histograms of the spatial periods L and heights h of the first and second orders. Statistics—n: 150 profiles from 5 cells; ∗P < 0.05; ∗∗P < 0.01; ∗∗∗P < 0.001; ∗∗∗∗P < 0.0001; ns: not significantly (Mann–Whitney test). Figure S2: imaging of dried neutrophil by CLSM. (a) Neutrophil, dried at room temperature in air. (b) Lateral projection of neutrophils dried at room temperature in air. Image captured using Zeiss Airyscan module. Channels: blue (Hoechst33342)—DNA, green (phalloidin + A488)—F-actin, and red (WGA + A594)—membrane. The image shows the spatial position of the nuclei, membrane, and F-actin. The difference is seen in the position of the membrane and F-actin. F-actin occupies a smaller portion of the membrane area and is colocalized with the nuclei. Scale bar = 5 μm. Figure S3: evaluation of the parameters of the nanostructure of the actin cytoskeleton of neutrophils. (a) Maxima of neutrophil actin fluorescence. 1—neutrophil dried at room temperature in air, 2—neutrophil fixation with GA0.5, and 3—neutrophil fixation with MeOH10. The top image is the whole cell. Scale bar = 5 μm. The bottom image is a 3 × 3 μm2 region with marked maxima of actin fluorescence. Scale bar = 1 μm. (b) The nearest neighbor distance analysis for F-actin in neutrophils. N = 3 cells. 25 points from each cell. ∗∗P < 0.01; ∗∗∗∗P < 0.0001 (Mann—Whitney test). (c) A table of the nearest neighbor distance values; mean ± SD. Table S1: biomechanical parameters and characteristics of the nanostructure of neutrophils and RBC under the action of modifiers. Table S2: the colocalization coefficients for neutro [file 1140176.f1.docx]

https://cloud.mail.ru/public/v2eJ/kqN9WCuqJ
